# Identification of biomarkers of male infertility through the circRNA expression profiling of seminal plasma

**DOI:** 10.7555/JBR.38.20240192

**Published:** 2025-05-20

**Authors:** Zhaode Liu, Xinrui Li, Xiaoyu Yang, Bohang Zhang, Dingdong Chen, Yan Yuan, Yiqiang Cui

**Affiliations:** 1 State Key Laboratory of Reproductive Medicine and Offspring Health, Nanjing Medical University, Nanjing, Jiangsu 211166, China; 2 State Key Laboratory of Reproductive Medicine and Offspring Health, Clinical Center of Reproductive Medicine, the First Affiliated Hospital of Nanjing Medical University, Nanjing, Jiangsu 210029, China

**Keywords:** seminal plasma, circular RNA, male infertility, RNA-seq

## Abstract

Circular RNAs (circRNAs) are key regulators of reproductive biology. However, limited information is available regarding circRNA expression profiles in seminal plasma samples from individuals with male infertility. The present study aimed to identify circRNAs associated with infertility in seminal plasma samples and to clarify their potential as biomarkers, as well as the possible molecular mechanisms underlying their functions. Next-generation RNA sequencing was conducted to analyze circRNA profiles in seminal plasma from healthy controls, oligoasthenospermia (OAZ) patients, and non-obstructive azoospermia (NOA) patients. Bioinformatics analysis revealed that 637 circRNAs were differentially expressed between OAZ and control subjects, and 272 circRNAs were differentially expressed between NOA and control subjects. The expression of key circRNAs (*hsa-SAP130_0002*, *hsa-TRPC1_0001*, *hsa-FBRS_0001*, *hsa-ACACA_0025*, *hsa-UTRN_0042*, and *hsa-ZNF532_0023*) was then validated by qPCR, and their diagnostic accuracy for infertility was confirmed through receiver operating characteristic curve analysis. Additionally, a possible circRNA-miRNA-mRNA regulatory network was constructed for these candidate biomarkers. Collectively, this study identifies a novel set of circRNAs with potential as diagnostic biomarkers for male infertility and provides molecular insights that may facilitate both diagnostic and therapeutic efforts.

## Introduction

Conception difficulties affect roughly one-seventh of childbearing couples, with male infertility accounting for half of all infertility cases. In many cases, the cause of male infertility is considered idiopathic, resulting in the limited treatment options available^[1–2]^. The current diagnosis of male infertility relies on semen analyses, *in vitro* testing, endocrine function evaluation, ultrasound scans, and testicular sperm extraction^[3]^. Thus, there is an urgent need to establish new biomarkers that can facilitate a definitive diagnosis of male infertility.

Circular RNAs (circRNAs) are increasingly recognized as key regulators of physiological and pathological processes, including those related to human reproductive function^[4–6]^. circRNAs present in seminal plasma have been functionally linked to male infertility^[7–9]^. However, most research to date has relied on the analysis of testicular tissue or exosomal samples without subsequent validation in seminal plasma, potentially leading to the omission of certain circRNAs that are differentially abundant in seminal plasma.

Generally, seminal plasma exhibits a low RNA concentration (approximately 1.75 mg/L) and poor RNA integrity, with a 28S/18S ratio of approximately 0.6^[10]^. For these reasons, seminal plasma-derived RNA is not well-suited for traditional approaches to library construction, which generally rely on several rounds of purification and other steps, potentially resulting in significant loss of sample. The SHERRY library construction technique overcomes these issues because it is a one-tube approach that allows for the efficient and streamlined processing of samples while minimizing pipetting loss, making it particularly suitable for analyzing RNAs present at low abundance^[11]^. In the present study, we employed the SHERRY method with random primers to achieve a reliable capture of circRNAs from seminal plasma.

In the present study, we compared seminal plasma circRNA profiles among patients with oligoasthenospermia (OAZ), non-obstructive azoospermia (NOA), and healthy control subjects, aiming to identify potential circRNA biomarkers associated with infertility in seminal plasma and to explore the possible molecular mechanisms underlying their functions.

## Materials and methods

### Sample collection

Samples of semen from healthy controls, OAZ, and NOA patients were collected at the Reproductive Medical Center of Jiangsu Provincial People's Hospital in accordance with the World Health Organization (WHO) manual for the examination of human semen and sperm quality (5th edition). OAZ was defined as a reduction in sperm concentration (< 15 million/mL) and either sperm progressive motility (< 32%) or total motility (< 40%) based on the WHO 2010 criteria (5th edition). Semen samples from NOA patients were considered azoospermic after at least three semen analyses, including centrifugation at 900 *g* for 15 min and microscopic examination of the sediment without the detection of sperm. Exclusion criteria included: (1) congenital abnormalities (Klinefelter syndrome, hypergonadotropic hypogonadism, Y chromosome microdeletions, *etc.*); (2) secondary diseases (mumps, reproductive system infections, testicular trauma, and patients who had undergone testicular surgery); and (3) obstructive azoospermia (normal testicular volume and serum FSH levels with obstructions found *via* reproductive system ultrasound). Samples of donor semen were collected from excess semen samples used for routine semen analyses. All subjects provided informed consent for sample use and were of Han Chinese ethnicity. The study was approved by the Institutional Review Board of Nanjing Medical University (Approval No. 2023-SZ-1) and conducted in accordance with the guidelines of the Declaration of Helsinki. After liquefaction at 37 ℃ for 30 min, semen samples were stored at −80 ℃.

### circRNA library construction

We extracted total RNA from the testes of three adult ICR mice using Trizol (Cat. #15596018CN, Invitrogen, Carlsbad, CA, USA). Subsequently, 8 µg of RNA was treated with 3 U/µg RNase R (Cat. #RNR07250, Lucigen, Middleton, WI, USA) at 37 ℃ for 10 min to eliminate linear RNA, followed by purification of the remaining RNA using VAHTS RNA Clean Beads. Next, rRNA was depleted from this purified RNA using a Ribo-off rRNA Depletion Kit (Human/Mouse/Rat) (Cat. #NR406, Vazyme, Nanjing, China), followed by a second round of purification using VAHTS RNA Clean Beads. These purified RNA samples were used for circRNA library preparation using a TruePrep RNA Library Prep Kit for Illumina (Cat. #TR502, Vazyme) as follows: (1) RNA denaturation; (2) reverse transcription; (3) tagmentation of DNA-RNA hybrid strands using Tn5 transposase; (4) amplification by PCR; and (5) purification.

Samples of post-thaw seminal plasma were initially centrifuged at 1600 *g* for 10 min and then at 12000 *g *for 10 min, followed by RNA extraction from these samples using Trizol LS (Cat. #10296010, Invitrogen). These samples were not subjected to RNase R treatment; instead, library preparation was conducted directly using the TruePrep RNA Library Prep Kit for Illumina. During Tn5 enzyme digestion, the enzyme was diluted 10-fold before use.

Following magnetic bead-based purification, the libraries were transferred to the Nanjing Jiangbei New Area Biomedicine Public Service Platform. Quality control analyses of these libraries were conducted using a LabChip before 150-bp paired-end sequencing. Data quality control was performed using FastQC. We conducted circular RNA sequencing on a total of 16 control seminal plasma samples, 15 OAZ seminal plasma samples, and 13 NOA seminal plasma samples. The study was approved by the Institutional Review Board of Nanjing Medical University (Approval No. 2307030-1).

### circRNA sequencing analysis

Following sequencing, all paired-end reads were extracted. Adapter removal and filtering of low-quality reads were performed with FASTp (v0.23.2), while rRNA sequences were eliminated with SortMeRNA (v4.3.4). The resultant reads were aligned to the GRCh38 human genome using Bowtie2 (v2.4.2). circRNAs were identified using the CIRI2, CIRCexplorer2, and Find_circ tools^[12–14]^. Algorithm-dependent variability in the detection of differentially expressed circRNAs was controlled by cross-validating the results from these tools. Only circRNAs detected by at least two of these three programs, with an average read count exceeding two, were retained for downstream analysis. circRNA annotation was then conducted using the CircAtlas 3.0 database (https://ngdc.cncb.ac.cn/circatlas).

### circRNA differential expression and functional annotation analysis

Differentially expressed circRNAs were identified using DESeq2 (v1.36.0) based on the established criteria, *i.e.*, |log_2_(fold change)| > 2 and *P* < 0.01. The functional roles of these circRNAs were predicted through Gene Ontology (GO) analyses (http://www.geneontology.org), analyzing the biological processes, cellular components, and molecular functions related to the target genes of these circRNAs. Significant enrichment was defined as *P* < 0.05.

### Quantitative reverse transcription-PCR (RT-qPCR)

The Hifair Ⅲ 1st Strand cDNA Synthesis Kit (Cat. #11139ES60, YEASEN, Shanghai, China) was used to reverse transcribe RNA from seminal plasma samples. circRNA sequences were obtained from CircAtlas 3.0, while primer design was performed using NCBI tools (https://www.ncbi.nlm.nih.gov). Primers for these circRNAs were developed based on the back-splicing junctions or spanning junction sites (***Supplementary Table 1***, available online). Agarose gel electrophoresis and Sanger sequencing were used to validate the amplification products from PCR. The Hieff qPCR SYBR Green Master Mix (Cat. #11202ES08, YEASEN) was used for qPCR analyses with the following settings: 95 ℃ for 5 min; 40 cycles of 95 ℃ for 10 s, and 60 ℃ for 30 s. The 2^−ΔΔCT^ method was used to calculate the relative expression level of each circRNA, with *GAPDH* as the normalizing control.

### circRNA-miRNA-mRNA interaction analysis

The interactions among circRNAs, miRNAs, and mRNAs were predicted using the StarBase (https://starbase.sysu.edu.cn), TargetScan (www.targetscan.org), and miRDB (http://mirdb.org) databases to investigate the functions of five circRNAs. The intersection of miRNA-mRNA targeting relationships predicted by both TargetScan and miRDB was identified, and the ceRNA regulatory network was subsequently visualized using Cytoscape (v3.10.1).

### Statistical analysis

Data were analyzed using GraphPad Prism 6 and were reported as the mean ± standard deviation or standard error of the mean. Findings were compared using Student's *t*-tests, with *P* < 0.05 considered statistically significant. Receiver operating characteristic (ROC) curves were employed to assess the diagnostic utility of circRNA expression levels.

## Results

### circRNA library preparation

**Figure 1:**
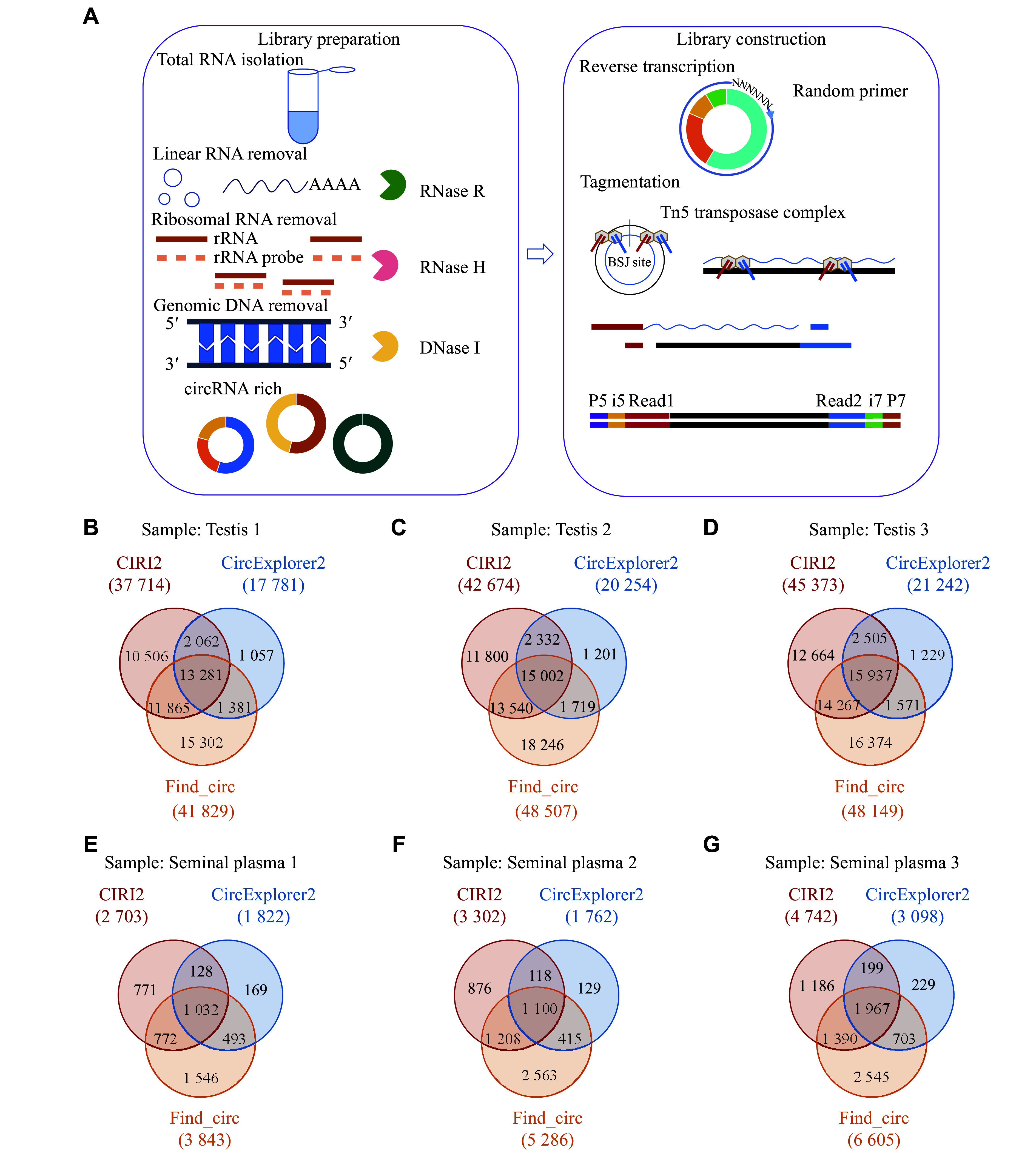
SHERRY method-based circRNA library construction. A: Overview of the process of circRNA library construction. B–G: Numbers of testis (B–D) and seminal plasma (E–G) circRNAs detected using CIRI2, CircExplorer2, and Find_circ. Abbreviation: BSJ, backsplice junction.

As circRNAs lack a poly-A tail, the oligo-dT primers used in the SHERRY method were substituted with random primers to facilitate circRNA capture during reverse transcription. We conducted a pilot analysis using testes from three adult ICR mice, and identified 13281, 15002, and 15937 circRNAs in the three murine testis samples, respectively (***Fig. 1A***–***1D***).

The LabChip system was used for quality control analysis of prepared libraries, revealing a peak fragment size of approximately 180 bp and an average library size of approximately 410 bp (***Supplementary Fig. 1A***–***1C***, available online). Approximately 95% and 90% of the raw data achieved Q20 and Q30 base quality scores, respectively. GC content in these samples was approximately 50%, while less than 1% of reads aligned to rRNA. These results confirmed the high quality of the circRNA sequencing data from murine testes.

To determine whether this library construction strategy results in significant differences in the numbers of identified circRNAs relative to more traditional approaches, we performed preliminary comparisons between the present results and those from other studies that employed different species, varied sample qualities, different sequencing data volumes, and distinct identification criteria. Studies have reported 15996 circRNAs in normal human testes^[6]^, 17094 in testis samples from NOA patients^[9]^, and 15201 in the testes of C57BL/6 mice^[15]^. These numbers are comparable to the circRNA counts identified in the present study, suggesting that this random primer-based SHERRY library construction strategy may identify a similar number of circRNAs while expediting the process of library construction, compared with conventional approaches.

Next, we applied this random primer-based SHERRY method to the construction of a seminal plasma circular RNA library and subsequently identified 1032, 1100, and 1967 circular RNAs by taking the intersection of the results from three tools in three seminal plasma samples, respectively (***Fig. 1E***–***1G***). The LabChip system was used for quality control analysis of prepared libraries, revealing a peak fragment size of approximately 180 bp and an average library size of approximately 340 bp (***Supplementary Fig. 1D–1F***).

Collectively, we have successfully applied the random primer-based SHERRY method to circular RNA library construction, facilitating subsequent research.

### Seminal plasma circRNA characteristics

**Figure 2:**
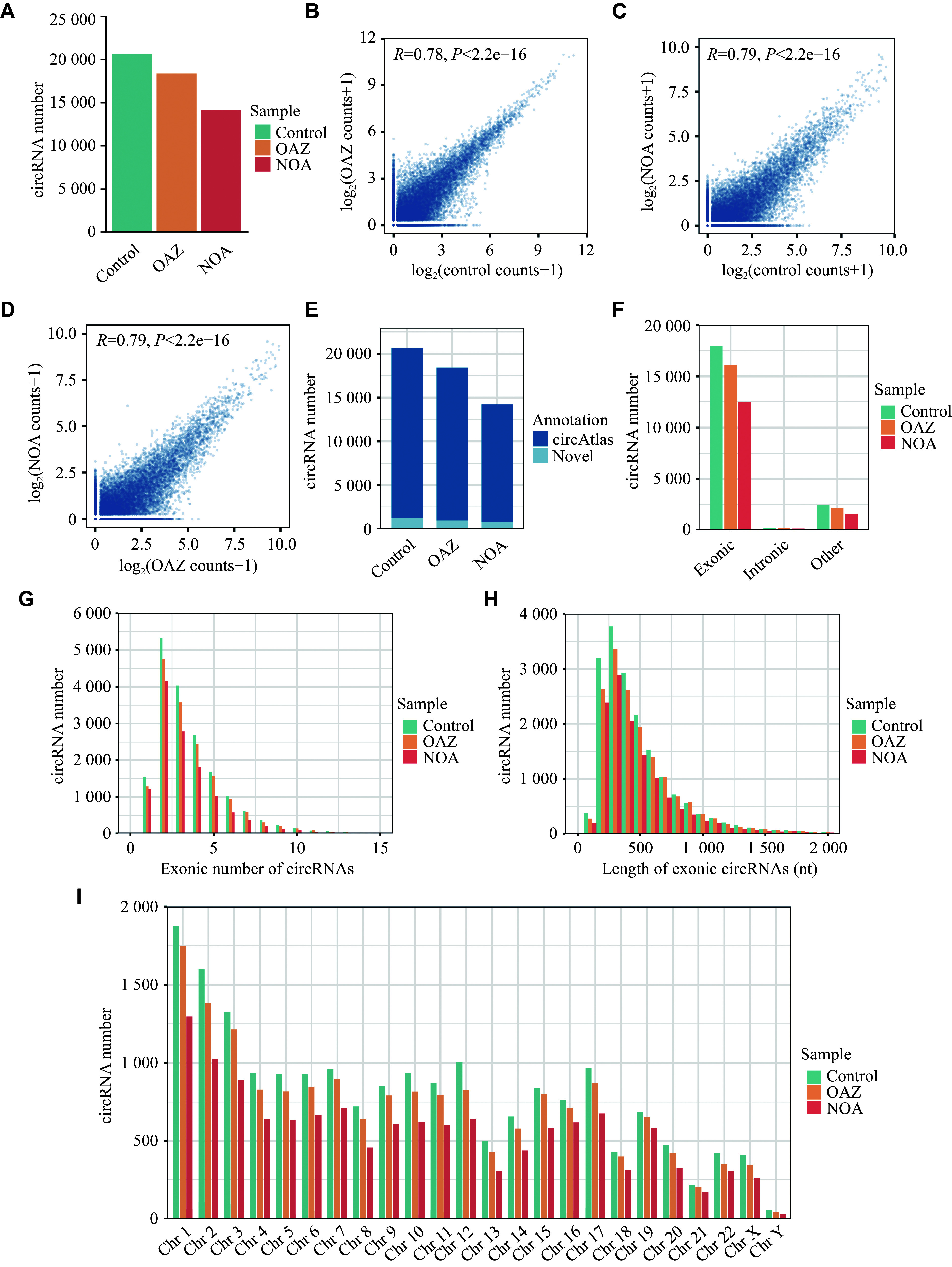
Characterization of the circRNAs present in human seminal plasma. A: Seminal plasma circRNAs in the healthy control (*n* = 16), OAZ (*n* = 15), and NOA (*n* = 13) groups. B–D: Pearson's correlation scatter plots for the expression of circRNAs between OAZ patients and controls (B), NOA patients and controls (C), and OAZ patients and NOA patients (D). E: Using CircAtlas 3.0, 19377, 17445, and 13438 known and 1267, 985, and 770 novel circRNAs were identified in the healthy control, OAZ, and NOA groups, respectively. F: Distributions of circRNAs derived from exonic regions in the healthy control, OAZ, and NOA groups. G: Numbers of back-spliced exons comprising the circRNAs identified in the indicated groups. H: circRNA length distributions in the indicated groups. I: circRNA chromosomal distributions. Abbreviations: OAZ, oligoasthenospermia; NOA, non-obstructive azoospermia.

Following the validation of the experimental approach, we established circRNA libraries using samples from 15 OAZ patients, 13 NOA patients, and 16 healthy subjects (***Table 1***) for sequencing. A total of 20644, 18430, and 14208 circRNAs were identified in seminal plasma samples from the control, OAZ, and NOA groups, respectively (***Fig. 2A***). We then conducted correlation analyses of these circRNAs to determine the relationships among the groups, and found a Pearson correlation coefficient (*R*-value) of 0.78 (OAZ *vs.* control; ***Fig. 2B***), 0.79 (NOA *vs.* control; ***Fig. 2C***), and 0.79 (NOA *vs.* OAZ; ***Fig. 2D***). The results suggest that all three groups exhibit generally similar circRNA profiles, supporting the reliability of these data.

**Table 1:** Participant characteristics

Variables	HC (*n*=16)	OAZ (*n*=15)	NOA (*n*=13)	*P*-value^a^	*P*-value^b^
Age (years)	31.00±3.83	32.67±6.34	29.62±3.80	0.7151	0.5634
Sperm concentration (10^6^/mL)	117.00±74.05	7.027±5.25	0	<0.001	<0.001
Progressive motility (%)	49.53±9.53	17.95±10.97	0	<0.001	<0.001
Sperm viability (%)	67.43±10.55	26.61±15.92	0	<0.001	<0.001
Total sperm number (10^6^/ejaculate)	358.40±215.30	16.66±10.46	0	<0.001	<0.001
Data are shown as mean ± standard deviation.^a^HC *vs.* OAZ patients.^b^HC *vs.* NOA patients.Abbreviations: HC, healthy controls; OAZ, oligoathenospermia; NOA, non-obstructive azoospermia.					

Subsequently, we compared the identified circRNAs using CircAtlas 3.0 to identify novel circRNAs, and found that 19377 (93.9%), 17445 (94.7%), and 13438 (94.6%) of the circRNAs in these three groups were known, whereas 1267 (6.1%), 985 (5.3%), and 770 (5.4%) were newly identified in human seminal plasma samples (***Fig. 2E***). These novel circRNAs may serve as potential molecular biomarkers for male infertility.

circRNAs are generated by splicing events that produce circular transcripts^[16]^. Therefore, we performed an alignment analysis of circRNAs identified in seminal plasma to genomic sequences. The results showed that in the control, OAZ, and NOA groups, these circRNAs primarily originated from exonic circularization, with counts of 17946 (86.9%), 16107 (87.4%), and 12504 (88.0%), respectively, followed by intronic sequences, with counts of 2477 (12.0%), 2154 (11.7%), and 1573 (11.1%), respectively, and genomic sequences, with counts of 221 (1.1%), 169 (0.9%), and 131 (0.9%), respectively (***Fig. 2F***). These findings align with the consensus that circRNAs primarily originate from exon splicing^[17]^.

We also assessed the number of exons comprising the detected circRNAs. The results showed that 12064 (58.4%), 10792 (58.6%), and 8756 (61.6%) in the control, OAZ, and NOA groups, respectively, contained two to four exons, while some circRNAs contained one exon, and the maximum number of exons was 15 (***Fig. 2G***). These exonic circRNAs were further classified into 100-nucleotide (nt) bins, with the majority of them being 200–500 nt in length (***Fig. 2H***), aligning with third-generation sequencing results from full-length circRNA libraries, with most circRNAs tending to be relatively short^[18]^. Statistical analysis of their chromosomal origins revealed that these circRNAs were distributed across all autosomes and both the X and Y chromosomes, although the number derived from the Y chromosome was lower than that from the other chromosomes (***Fig. 2I***).

Together, these findings highlight the diversity of seminal plasma circRNAs in healthy controls, OAZ patients, and NOA patients. Thus, these circRNAs may be used for downstream analysis to explore biomarkers related to male infertility.

### Analyses of the differential expression and functional enrichment of circRNAs

**Figure 3:**
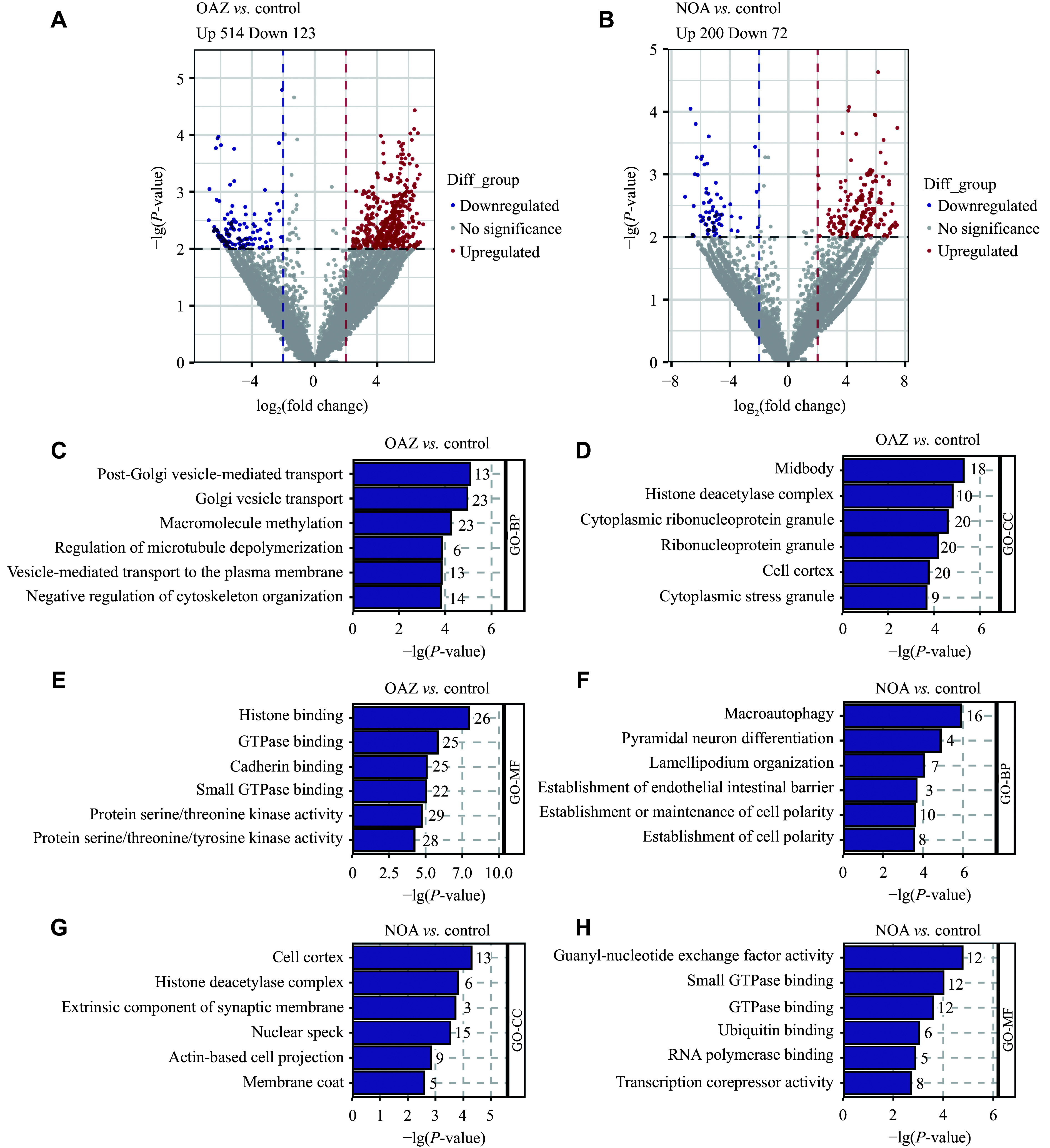
Identification and analysis of circRNAs differentially expressed between male infertility patients and controls. A and B: Volcano plots corresponding to circRNAs differentially expressed in the seminal plasma of OAZ (*n* = 15) and control (*n* = 16; A) and NOA (*n* = 13) and control (*n* = 16; B) patients. Red and blue points indicate significantly upregulated and downregulated circRNAs, respectively, while circRNAs in gray did not differ significantly. C–H: Gene ontology enrichment analysis of parental genes associated with differentially expressed circRNAs between male infertility patients and controls. Biological processes (BP; C and F), cellular components (CC; D and G), and molecular functions (MF; E and H) terms enriched between the NOA or OAZ and control groups are shown. Abbreviations: OAZ, oligoasthenospermia; NOA, non-obstructive azoospermia.

To investigate the functions of circRNAs in male infertility, we initially screened the differentially expressed circRNAs between the control group and the OAZ or NOA groups [|log_2_(fold change)| > 2, *P* < 0.01] by volcano plot analysis. In total, 637 circRNAs were differentially expressed between the OAZ and control groups (514 upregulated, 123 downregulated; ***Fig. 3A***), while 272 were differentially expressed between the NOA and control groups (200 upregulated, 72 downregulated) (***Fig. 3B***).

circRNAs exhibit substantial sequence consistency with their linear RNA counterparts and have been shown to affect parental gene expression by regulating transcription initiation and elongation^[19–21]^. Accordingly, we performed GO analysis of parental genes associated with differentially expressed circRNAs between the OAZ and control groups. The results showed that enriched biological processes were significantly related to post-Golgi vesicle-mediated transport and Golgi vesicle transport (***Fig. 3C***). Notably, the mutation (L967Q) in vacuolar protein sorting-associated protein 54 (*Vps54*), a differentially expressed circRNA parental gene, may contribute to defective functions of vesicles that are important for sperm acrosome formation, thus impairing acrosome development and causing sperm cells to remain spherical^[22]^. Enriched cellular component terms were mostly related to the midbody and histone deacetylase complexes (***Fig. 3D***). Knockdown of *Mark4*, a differentially expressed circRNA parental gene, impairs the permeability of tight junctions at the Sertoli cell blood-testis barrier and interferes with the cytoskeletal arrangement of actin and microtubules in Sertoli cells^[23]^. Enriched molecular functions were significantly related to protein binding, such as histone binding, GTPase binding, and cadherin binding (***Fig. 3E***). The loss of NAD-dependent protein deacetylase sirtuin-1 (*Sirt1*; a differentially expressed circRNA parental gene) has been shown to alter post-translational histone modifications and interfere with the histone-to-protamine transition, resulting in the impairment of spermatogenesis in mice^[24]^. These findings suggest that differentially expressed circRNA parental genes in the OAZ group may be associated with impaired spermatogenesis.

We further performed GO analysis of parental genes associated with differentially expressed circRNAs between the NOA and control groups. The results showed that enriched biological processes were mostly related to cellular autophagy and the establishment of cell polarity (***Fig. 3F***). Mice lacking the E3 ubiquitin-protein ligase HUWE1 (*Huwe1*), a differentially expressed circRNA parental gene, exhibit Sertoli cell-only phenotypes^[25]^. Enriched cellular component terms were significantly related to the cell cortex and histone deacetylase complex (***Fig. 3G***). The E3 ubiquitin-protein ligase *Itchy*, a differentially expressed circRNA parental gene, mutant mice present with lower mature spermatid counts and reduced litter sizes compared with wild-type controls^[26]^. Enriched molecular functions were significantly related to protein binding, including GTPase binding, ubiquitin binding, and RNA polymerase binding (***Fig. 3H***). *Tcerg1l*, a differentially expressed circRNA parental gene, deficiency results in the production of spermatogonial stem cells that are unable to undergo normal differentiation under conditions of retinoic acid induction, maintaining high numbers of stem cells^[27]^. The differentially expressed circRNA parental genes in the NOA group may thus also be associated with impaired spermatogenesis.

### circRNAs have the potential to serve as candidate biomarkers of male infertility

**Figure 4:**
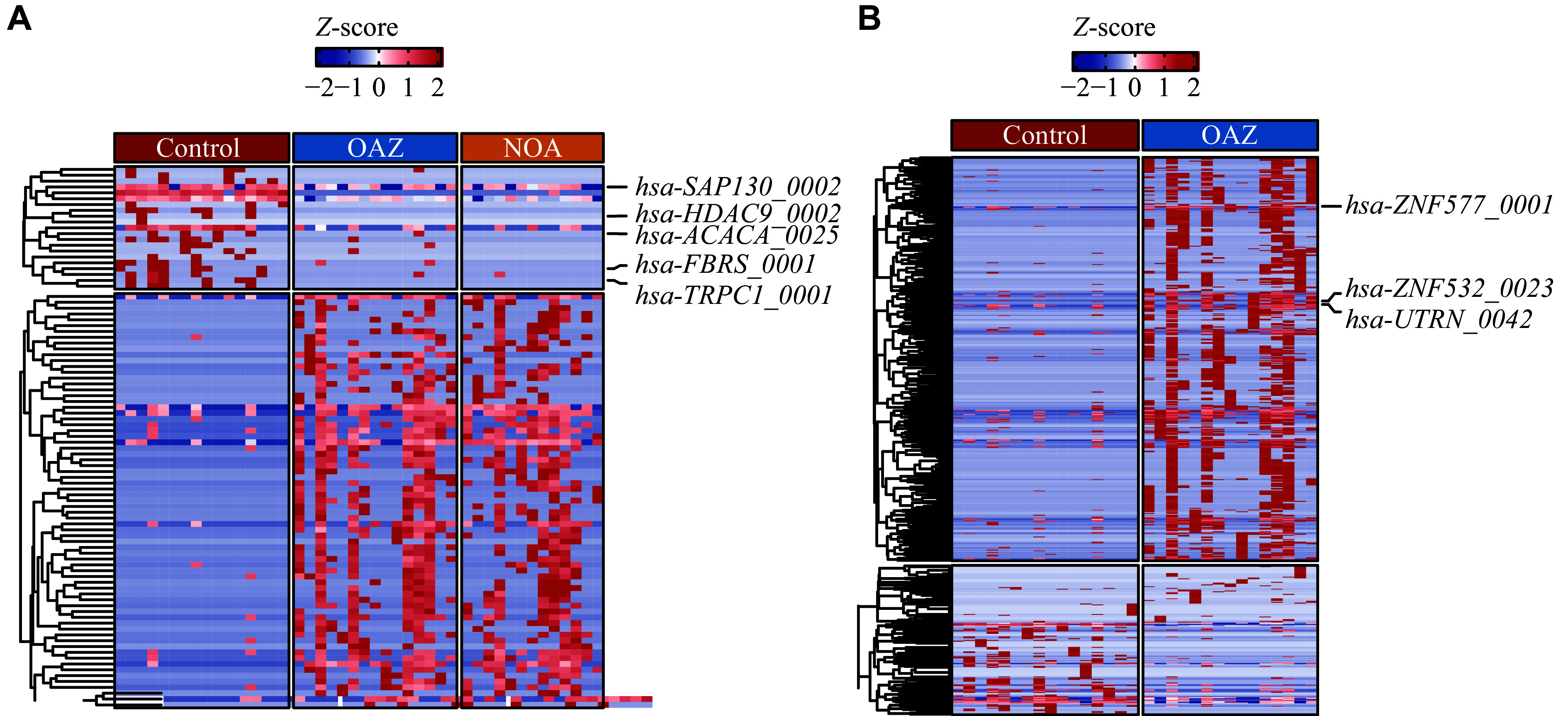
Selection and validation of homologous circRNAs as candidate biomarkers for male infertility. A: A heatmap showing the overlapping differentially expressed circRNAs in the male infertility and control groups was constructed. Control (*n* = 16), OAZ (*n* = 15), and NOA (*n* = 13). B: A heatmap showing the differentially expressed circRNAs in the oligoasthenospermia and the control group was constructed. Control (*n* = 16) and OAZ (*n* = 15). Abbreviations: OAZ, oligoasthenospermia; NOA, non-obstructive azoospermia.

To further explore the association between these circRNAs and male infertility, we constructed a heatmap of differentially expressed circRNAs based on the intersection between the OAZ *vs.* control and NOA *vs.* control groups, revealing 92 overlapping differentially expressed circRNAs (71 upregulated, 21 downregulated; ***Fig. 4A***). Of these, circRNAs potentially involved in normal function maintenance in healthy individuals were selected for further study. circRNA conservation has been a focus in recent years, enabling screening for cancers, reproductive phenotypes, and other conditions of interest^[9,28–29]^. Notably, conservation analysis of the 21 overlapping downregulated circRNAs using CircAtlas 3.0 revealed five circRNAs with homologous counterparts in humans, *Macaca mulatta*, and mice: *hsa-SAP130_0002*, *hsa-FBRS_0001*, *hsa-ACACA_0025*, *hsa-TRPC1_0001*, and *hsa-HDAC9_0002* (***Fig. 4A***). Additionally, three circRNAs differentially expressed in the OAZ and control groups were selected based on their fold-change, including *hsa-ZNF577_0001*, *hsa-UTRN_0042*, and *hsa-ZNF532_0023* (***Fig. 4B***). Of these, *hsa-ZNF577* is differentially expressed between prostate cancer and paracancerous tissues, suggesting its potential as a biomarker for prostate cancer^[30]^. *Utrn* deficiency in mouse models of muscular dystrophy results in delayed testicular development and reduced sperm vitality^[31]^, while *circUTRN* induces apoptosis in pancreatic cell lines^[32]^. Collectively, these findings indicate that these eight circRNAs may be candidates for further study.

### Validation of differentially expressed circRNAs by RT-qPCR

The above-mentioned eight candidate circRNAs, as well as five circRNAs selected at random from differentially expressed circRNAs (*hsa-ERCC2_0003*, *hsa-KIAA1549_0025*, *hsa-MUC16_0003*, *hsa-MUC16_0002*, and *hsa-SLCO2A1_0002*), were further validated by RT-PCR. Agarose gel electrophoresis and Sanger sequencing confirmed that the sequences of these circRNAs were accurate (***Supplementary Figs. 2*** and ***3***, available online). The results further confirmed the findings from the above bioinformatics analyses and suggest that these circRNAs are present in seminal plasma.

**Figure 5:**
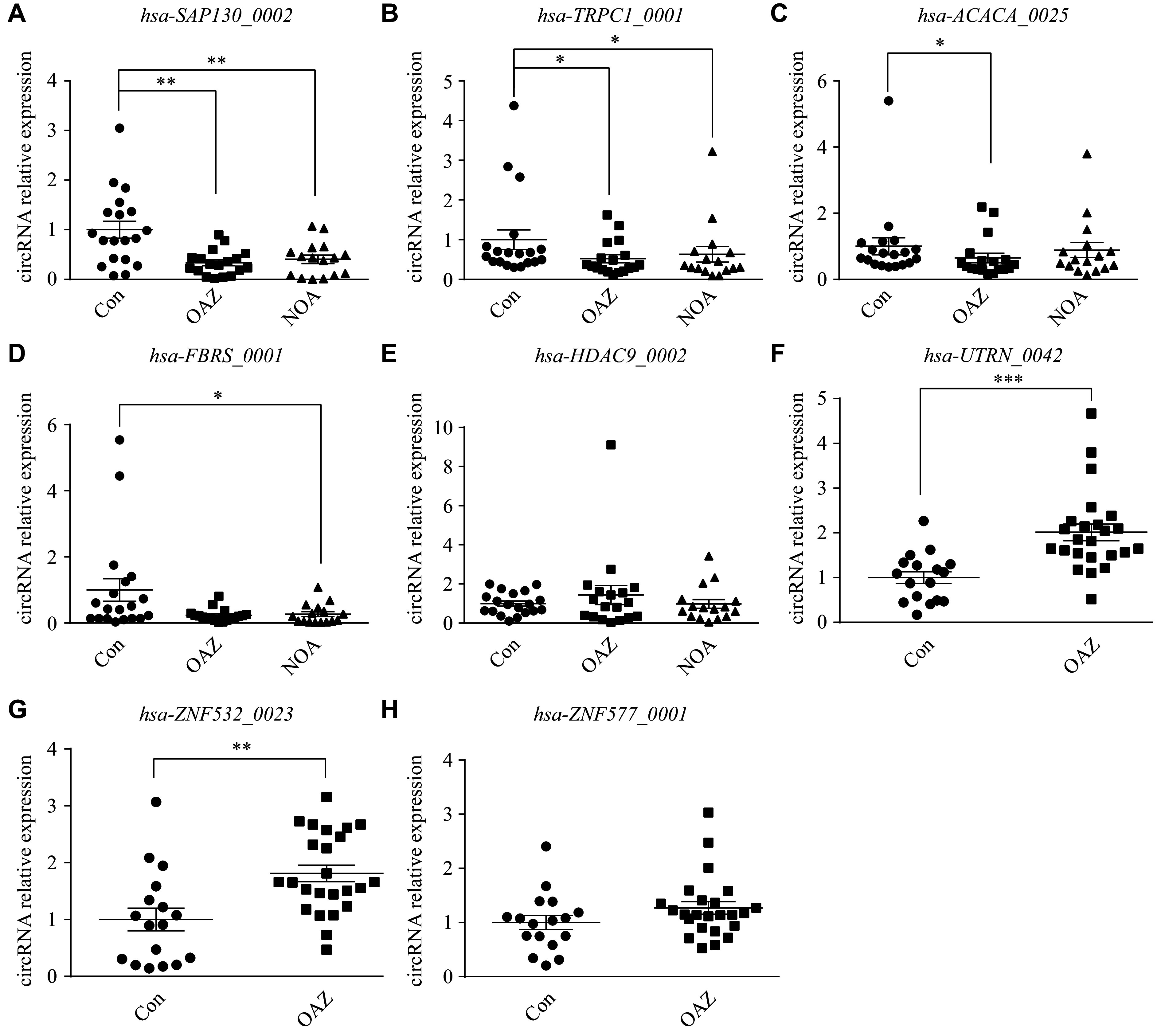
Quantitative reverse transcription-PCR (RT-qPCR) analysis of candidate circRNA biomarkers of male infertility. A–E: RT-qPCR analyses were performed to examine the expression levels of seminal plasma circRNAs in the control (Con), OAZ, and NOA groups. Comparisons of the relative expression levels of *hsa-SAP130_0002* (A), *hsa-TRPC1_0001* (B), *hsa-ACACA_0025* (C), *hsa-FBRS_0001* (D), and *hsa-HDAC9_0002* (E) in seminal plasma of the control (*n* = 19), OAZ (*n* = 18), and NOA (*n* = 16) groups. F–H: RT-qPCR analyses were performed to examine the expression levels of seminal plasma circRNAs in the control and OAZ groups. Comparisons of the relative expression levels of *hsa-UTRN_0042* (F), *hsa-ZNF532_0023* (G), and *hsa-ZNF577_0001* (H) in seminal plasma of the control (*n* = 17) and OAZ (*n* = 24) groups. Data are shown as mean ± standard error of the mean. ^*^P < 0.05, ^**^P < 0.01, and ^***^P < 0.001 by Student's *t*-test. Abbreviations: OAZ, oligoasthenospermia; NOA, non-obstructive azoospermia.

*hsa-SAP130_0002* levels were lower in the OAZ and NOA groups than in the control group (*P* < 0.01; ***Fig. 5A***), with *hsa-TRPC1_0001* showing a similar reduction (*P* < 0.05; ***Fig. 5B***). Moreover, *hsa-ACACA_0025* was significantly downregulated in the OAZ group, compared with the control group (*P* < 0.05), whereas only a downward trend was observed in the NOA group (***Fig. 5C***). Significantly reduced *hsa-FBRS_0001* levels were observed in the NOA group, compared with the control group (*P* < 0.05), with a non-significant decrease in the OAZ group (***Fig. 5D***). No significant differences in *hsa-HDAC9_0002* levels were observed between the groups (***Fig. 5E***).

Seminal plasma *hsa-UTRN_0042* levels were significantly increased in OAZ patients, compared with healthy controls (*P* < 0.001; ***Fig. 5F***), with *hsa-ZNF532_0023* showing a similar increase (*P* < 0.01; ***Fig. 5G***). Conversely, *hsa-ZNF577_0001* levels showed only an upward trend in the OAZ group, compared with the control group (***Fig. 5H***).

### Diagnostic potential of differentially expressed circRNAs in seminal plasma

To evaluate the potential of these circRNA biomarkers as candidates for distinguishing infertile individuals from healthy controls, we performed ROC curve analyses based on the levels of circRNA.

**Figure 6:**
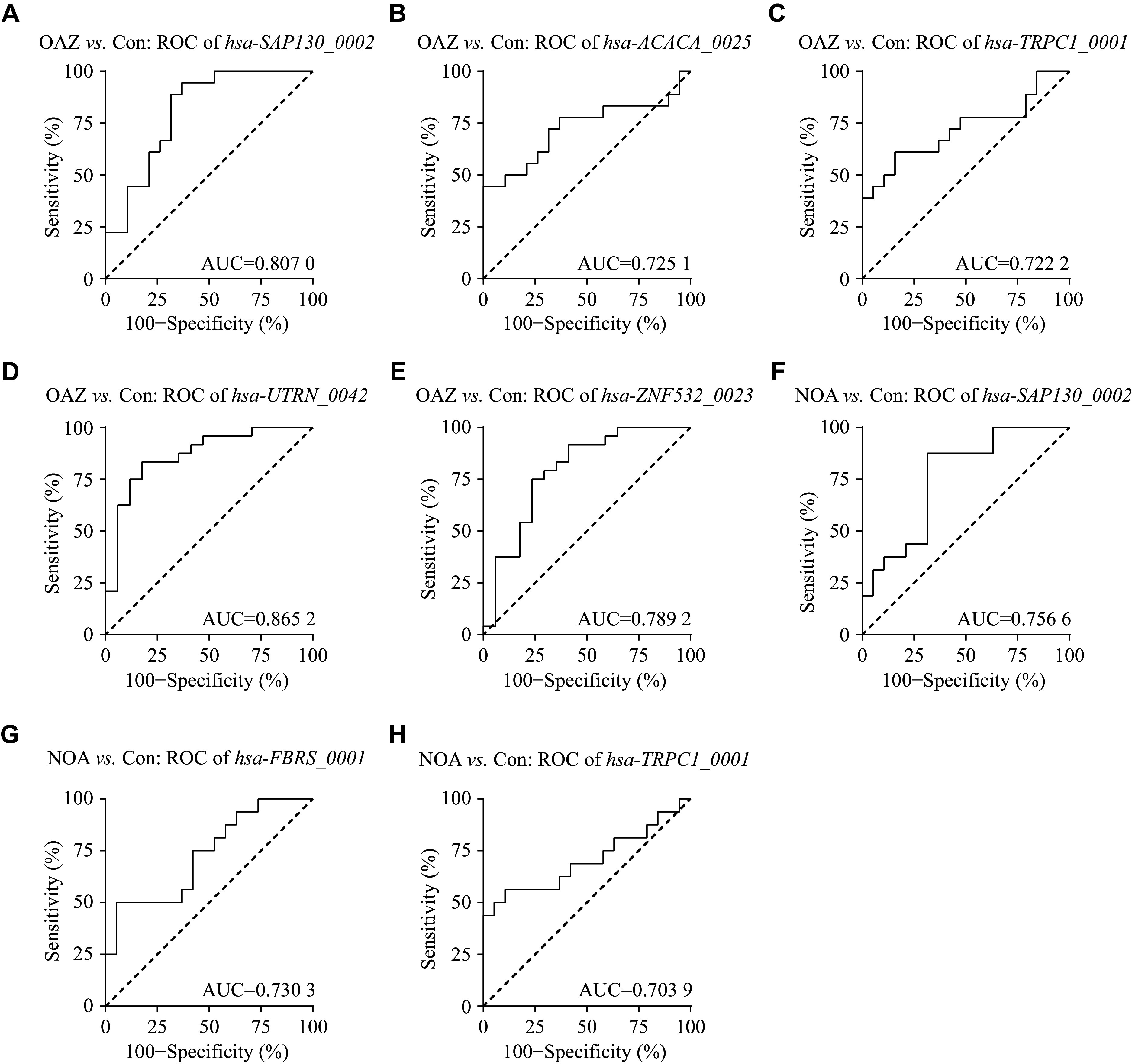
ROC analyses of circRNAs in the seminal plasma. Receiver operating characteristic curve analysis of differentially expressed circRNAs in seminal plasma. Abbreviations: Con, control; OAZ, oligoasthenospermia; NOA, non-obstructive azoospermia; AUC, area under curve.

*hsa-SAP130_0002* effectively distinguished OAZ patients from healthy controls with sensitivity and specificity of 88.89% and 68.42%, respectively (area under curve [AUC] = 0.8070, standard error [SE] = 0.07, 95% confidence interval [CI]: 0.6648–0.9492, cut-off = 0.686, *P* < 0.01; ***Fig. 6A***). Similarly, *hsa-TRPC1_0001* exhibited sensitivity and specificity of 61.11% and 84.21%, respectively (AUC = 0.7222, SE = 0.09, 95% CI: 0.5518–0.8927, cut-off = 0.396, *P* < 0.05; ***Fig. 6B***); *hsa-ACACA_0025* showed sensitivity and specificity values of 77.78% and 63.16%, respectively (AUC = 0.7251, SE = 0.09, 95% CI: 0.5521–0.8982, cut-off = 0.605, *P* < 0.05; ***Fig. 6C***); *hsa-UTRN_0042* demonstrated sensitivity and specificity of 83.33% and 82.35%, respectively (AUC = 0.8652, SE = 0.06, 95% CI: 0.7483–0.9821, cut-off = 1.395, *P* < 0.001; ***Fig. 6D***); and *hsa-ZNF532_0023* displayed sensitivity and specificity of 75% and 76.47%, respectively (AUC = 0.7892, SE = 0.08, 95% CI: 0.6391–0.9393, cut-off = 1.392, *P* < 0.01; ***Fig. 6E***). These results indicate that *hsa-SAP130_0002*, *hsa-TRPC1_0001*, *hsa-ACACA_0025*, *hsa-UTRN_0042*, and *hsa-ZNF532_0023* may serve as biomarkers of OAZ.

Additionally, circRNA *hsa-SAP130_0002* showed the potential to distinguish NOA patients from healthy controls with sensitivity and specificity of 87.50% and 68.42%, respectively (AUC = 0.7566, SE = 0.08, 95% CI: 0.5950–0.9182, cut-off = 0.716, *P* < 0.01; ***Fig. 6F***). Similarly, *hsa-TRPC1_0001* showed sensitivity and specificity of 56.25% and 89.47% (AUC = 0.7039, SE = 0.09, 95% CI: 0.5187–0.8889, cut-off = 0.351, *P* < 0.05; ***Fig. 6G***) and *hsa-FBRS_0001* exhibited sensitivity and specificity of 50% and 94.74% (AUC = 0.7303, SE = 0.09, 95% CI: 0.5623–0.8982, cut-off = 0.098, *P* < 0.05; ***Fig. 6H***). These results suggest that *hsa-SAP130_0002*, *hsa-TRPC1_0001*, and *hsa-FBRS_0001* may act as potential NOA-related biomarkers.

### circRNA-miRNA-mRNA network establishment

**Figure 7:**
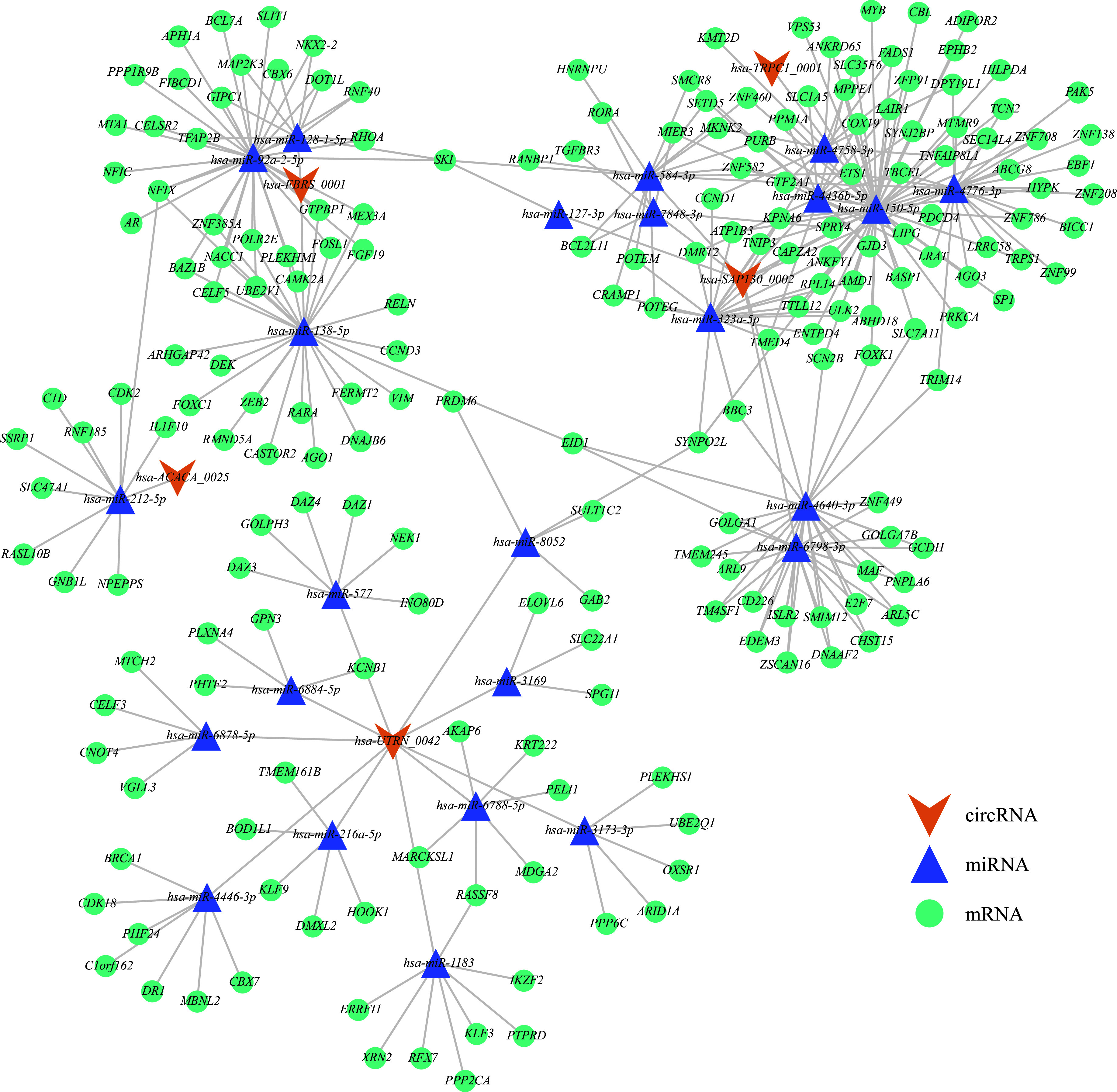
The circRNA-miRNA-mRNA network. A network comprising 24 miRNAs, 203 mRNAs, and 338 interactions was established by StarBase, TargetScan, and miRDB.

circRNAs act as key regulators of mRNA expression through their ability to bind to miRNAs competitively^[33]^. To investigate the mechanisms underlying their functions, we predicted miRNAs that potentially interact with the validated circRNAs using TargetScan. In total, 24 target miRNAs were identified, the targets of which were subsequently predicted with the StarBase and miRDB databases. We then established a network comprising five circRNAs, 24 miRNAs, 203 mRNAs, and 338 distinct interactions (***Supplementary Table 2***, available online). No interactions were predicted between *hsa-ZNF532_0023* and any target miRNAs. The predicted pathways were interconnected. For instance, *hsa-TRPC1_0001* and *hsa-SAP130_0002* both interacted with hsa-miR-150-5p. Multiple miRNAs may also target the same mRNA, as in the case of the *SKI* mRNA targeted by hsa-miR-92a-2-5p, hsa-miR-128-1-5p, hsa-miR-127-3p, and hsa-miR-584-3p (***Fig. 7***).

**Figure 8:**
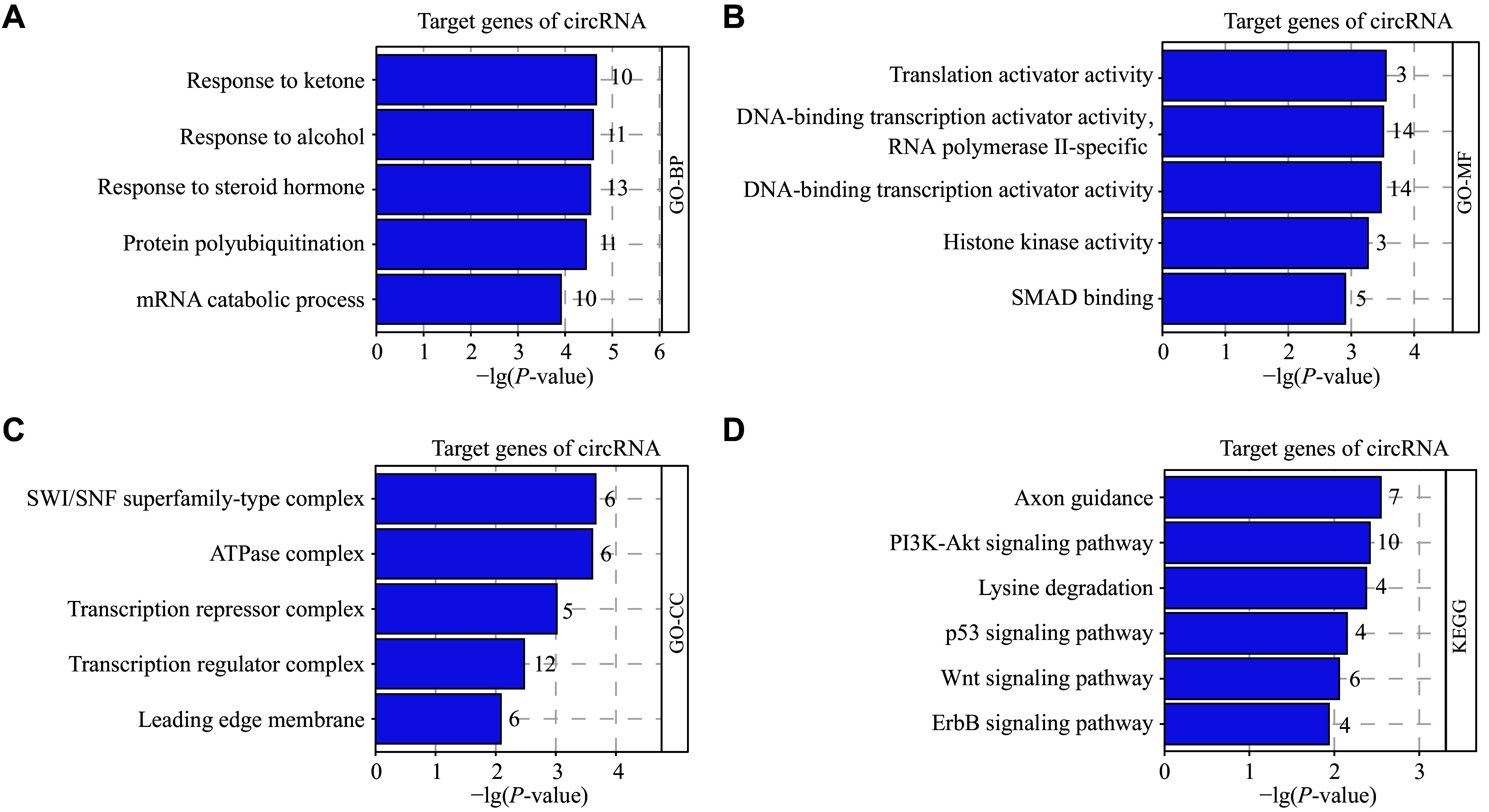
GO enrichment analysis and KEGG pathway analysis of circRNA target genes. A–C: GO enrichment analysis of circRNA target genes in the circRNA-miRNA-mRNA networks, including analyses of biological processes (A), molecular functions (B), and cellular components (C). D: The KEGG signaling pathways of circRNA target genes in the circRNA-miRNA-mRNA network. Abbreviations: GO, Gene Ontology; KEGG, Kyoto Encyclopedia of Genes and Genomes.

Subsequently, we performed GO analysis of the target gene downstream of these circRNAs. The results showed that these genes were primarily enriched in biological processes such as responses to ketones, alcohol, and steroid hormones (***Fig. 8A***). Mice that lack the androgen receptor fail to receive androgens, leading to meiotic arrest and male infertility^[34]^. These genes were also enriched in molecular functions, including transcription and translational activation (***Fig. 8B***). The loss of the deleted in azoospermia (*DAZ*) genes *DAZ1*, *DAZ3*, and *DAZ4* leads to spermatogenic arrest, contributing to male infertility^[35]^. The cellular component analysis revealed that the most enriched GO terms included the SWI/SNF superfamily complex and the ATPase complex (***Fig. 8C***). Male mice with a knockout of *Baz1a*, a protein responsible for chromatin remodeling, exhibit widespread gene expression abnormalities contributing to impaired spermatogenesis or azoospermia^[36]^. The genes cited above all belong to the circRNA-miRNA-mRNA network. The selected circRNAs are likely to competitively bind to miRNAs targeting these mRNAs, thereby interfering with mRNA expression and thus affecting spermatogenesis. These findings suggest that the identified circRNA target genes are critical to spermatogenesis and that seminal plasma biomarker circRNAs may regulate spermatogenesis by regulating specific mRNAs.

KEGG pathway analysis contributed to further understanding of circRNA biofunctions and revealed the major signal transduction and metabolic pathways associated with male infertility. The top pathways associated with seminal plasma circRNA target genes include 'axon guidance', 'PI3K-Akt signaling pathway', 'lysine degradation', 'p53 signaling pathway', 'Wnt signaling pathway', 'ErbB signaling pathway', *etc*. (***Fig. 8D***). Among these, the PI3K-Akt signaling pathway plays a crucial role in promoting the growth and survival of immature Sertoli cells and spermatogenic cells^[37]^. The Wnt signaling pathway is essential for male reproductive functions, with its various components related to various reproductive anomalies^[38]^. The majority of these pathways are linked to male infertility pathophysiology through their dysregulation, suggesting that circRNAs may regulate these functional networks in male infertility.

## Discussion

The present study aimed to explore the potential of circRNAs as biomarkers for male infertility. Given the established roles of circRNAs in spermatogenesis and their stability in seminal plasma, they represent promising candidates for non-invasive biomarkers. To this end, we conducted high-throughput sequencing to screen circRNA expression profiles in healthy controls, OAZ patients, and NOA patients. From this, we identified a subset of circRNAs associated with male infertility, which were subsequently validated as diagnostic biomarkers and potential therapeutic targets. These findings offer valuable insight into the molecular mechanisms underlying spermatogenesis and male infertility, providing a foundation for developing new fertility treatments.

While previous studies have explored the association between circRNAs and male infertility, they have primarily focused on testicular tissue^[39–40]^, which requires invasive procedures that are less suitable for large-scale studies. In contrast, our study employed a non-invasive approach using seminal plasma to examine the relationships between circRNA expression patterns and male infertility. Seminal plasma samples from 16 controls, 15 OAZ patients, and 13 NOA patients were collected and analyzed, revealing a large number of circRNAs. Through ROC curve analyses, strong correlative relationships were found between the levels of certain circRNAs in seminal plasma and male infertility, suggesting a potential mechanistic link between these circRNAs and the etiology of infertility.

Most circRNAs studied to date have been discovered and profiled through microarray^[39]^, next-generation sequencing^[7]^, and nanopore sequencing^[15]^. While next-generation sequencing enables accurate detection of short sequences, it requires substantial sequencing depth and is not well-suited for capturing full-length circRNAs. Meanwhile, nanopore sequencing can detect full-length circRNAs but lacks precision. In the present study, we used the SHERRY method as a novel approach to overcome these deficiencies by taking advantage of the RNA/DNA hybrid duplex that forms during reverse transcription. By using the Tn5 transposase to bind randomly and cleave these hybrid DNA-RNA chains, followed by PCR amplification of the fragmented cleavage products, the SHERRY method enables the rapid and straightforward construction of libraries from small amounts of RNA. We employed this strategy by combining random primers for circRNA sequencing and identified multiple differentially expressed circRNAs related to male infertility in seminal plasma samples from 44 subjects. Notably, we discovered six novel circRNAs. These circRNAs were subsequently validated *via* RT-qPCR.

circRNAs are characterized by their high abundance, stability, evolutionary conservation, and cellular specificity^[41]^. They are detectable in body fluids and tissues from patients, offering potential as valuable non-invasive biomarkers for diagnosis and prognosis, including the prediction of survival time^[42-43]^. Studies have demonstrated that circRNAs remain stable in seminal plasma when stored at room temperature for up to one day, with minimal risk of degradation^[6]^. This property enhances the feasibility of clinical research by preserving circRNA integrity during routine sample handling. In the present study, we identified a panel of circRNAs associated with male infertility. These circRNAs may serve as non-invasive biomarkers for combined diagnostics in clinical applications. In the future, these circular RNAs may be used to induce positive changes in infertile patients.

In the present study, we identified a panel of circular RNAs associated with male infertility, including *hsa-SAP130_0002*, *hsa-TRPC1_0001*, *hsa-FBRS_0001*, *hsa-ACACA_0025*, *hsa-UTRN_0042*, and *hsa-ZNF532_0023*, whose parental genes are predominantly expressed in the testes or prostate. Notably, these circRNAs did not show altered expression patterns in other diseases, as analyzed using the circRNADisease v2.0 database^[44]^. The circRNAs preferentially expressed in the testis and prostate are likely to enter sperm through exosomes and further affect spermatogenesis, sperm maturation, and sperm function by sponging miRNAs targeting these mRNAs^[7]^.

To further explore the pathophysiological mechanisms of male infertility, we constructed a circRNA-miRNA-mRNA regulatory network involving circRNAs, miRNAs, and their target genes, and performed GO enrichment analysis of the circRNA target genes. Some infertility-related genes were identified in these networks, including *DAZ* family genes (*DAZ1*, *DAZ3*, and *DAZ4*), NF1 family TF motifs (*NFIX*), and ras association domain family 8 (*RASSF8*). *DAZ* deficiency may lead to impaired multiplication of spermatogonia and failure in spermatogenesis^[45]^. *NFIX* suppression promotes cell proliferation and DNA synthesis but inhibits early apoptosis in human spermatogonial stem cells^[46]^. *RASSF8* inhibits spermatogonial stem cell proliferation *in vitro*^[47]^. Collectively, these results indicate that circRNAs may contribute to male infertility by competitively binding to miRNAs that target infertility genes.

While these analyses provide preliminary confirmation of the diagnostic performance of the identified circRNAs, the molecular mechanisms by which they influence fertility remain to be established. Future studies should use model cell lines and other experimental systems to gain direct insight into the mechanistic functions of these circRNAs. Specifically, validating the circRNA-miRNA-mRNA pathways predicted in the present study will be critical to clarify the regulatory importance of circRNAs as factors that shape male reproductive outcomes and the therapeutic potential of these circRNAs.

## SUPPLEMENTARY DATA

Supplementary data to this article can be found online.
