# Supplementary material for: Identification of biomarkers of male infertility through the circRNA expression profiling of seminal plasma
Source: J Biomed Res. 2025 May 20;39(4):367–81. doi: 10.7555/JBR.38.20240192 (PMC12336409; doi:10.7555/JBR.38.20240192)
Supplement: Supplementary file 1 — Supplementary data to this article can be found online. [file jbr-39-4-367-Supplementary.pdf]

Available online at www.jbr-pub.org.cn

Open Access at PubMed Central

Journal of Biomedical Research, 2025 39(4): 1–6

*Supplementary Data*

JBR

# Identification of biomarkers of male infertility through the circRNA expression profiling of seminal plasma

Zhaode Liu[1,△], Xinrui Li[1,△], Xiaoyu Yang[2], Bohang Zhang[1], Dingdong Chen[1], Yan Yuan[1,✉], Yiqiang Cui[1,✉]

[1]*State Key Laboratory of Reproductive Medicine and Offspring Health, Nanjing Medical University, Nanjing, Jiangsu 211166, China;*
[2]*State Key Laboratory of Reproductive Medicine and Offspring Health, Clinical Center of Reproductive Medicine, the First Affiliated Hospital of Nanjing Medical University, Nanjing, Jiangsu 210029, China.*

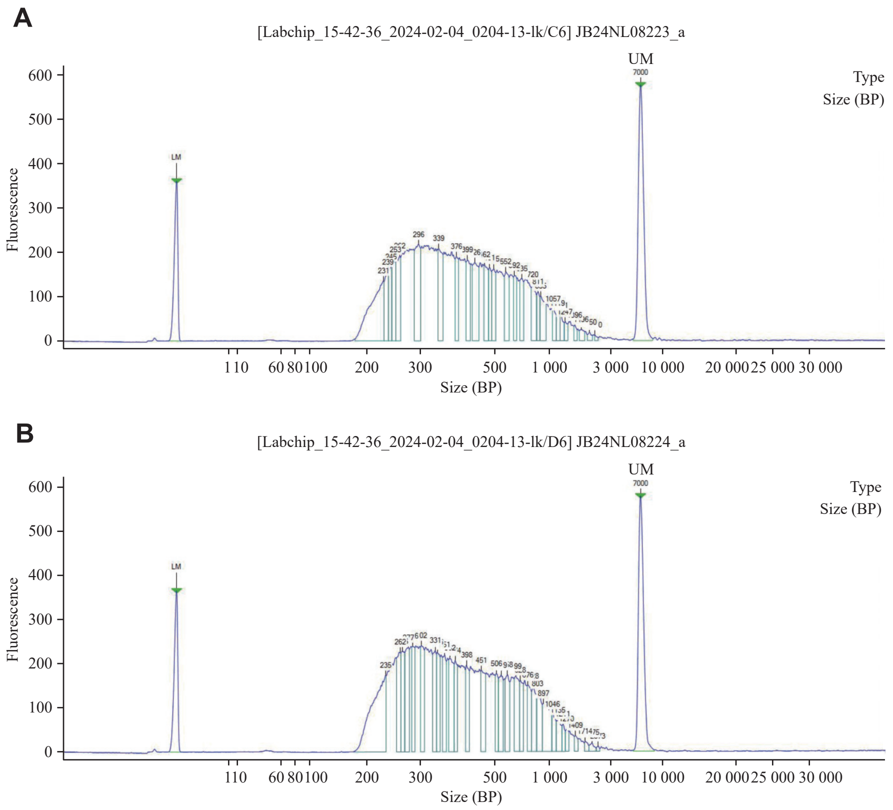


(Continued)

△These authors contributed equally to this work.

✉Corresponding authors: Yan Yuan and Yiqiang Cui, State Key Laboratory of Reproductive Medicine and Offspring Health, 101 Longmian Avenue, Nanjing Medical University, Nanjing, Jiangsu 211166, China. E-mails: yuanyan@njmu.edu.cn (Yuan) and cuiyiqiang@126.com (Cui).

Received: 03 July 2024; Revised: 23 April 2025; Accepted: 28 April 2025; Published online: 20 May 2025

CLC number: R698.2, Document code: A

The authors reported no conflict of interests.

This is an open access article under the Creative Commons Attribution (CC BY 4.0) license, which permits others to distribute, remix, adapt and build upon this work, for commercial use, provided the original work is properly cited.

© 2025 by Journal of Biomedical Research.

https://doi.org/10.7555/JBR.38.20240192

(Continued)

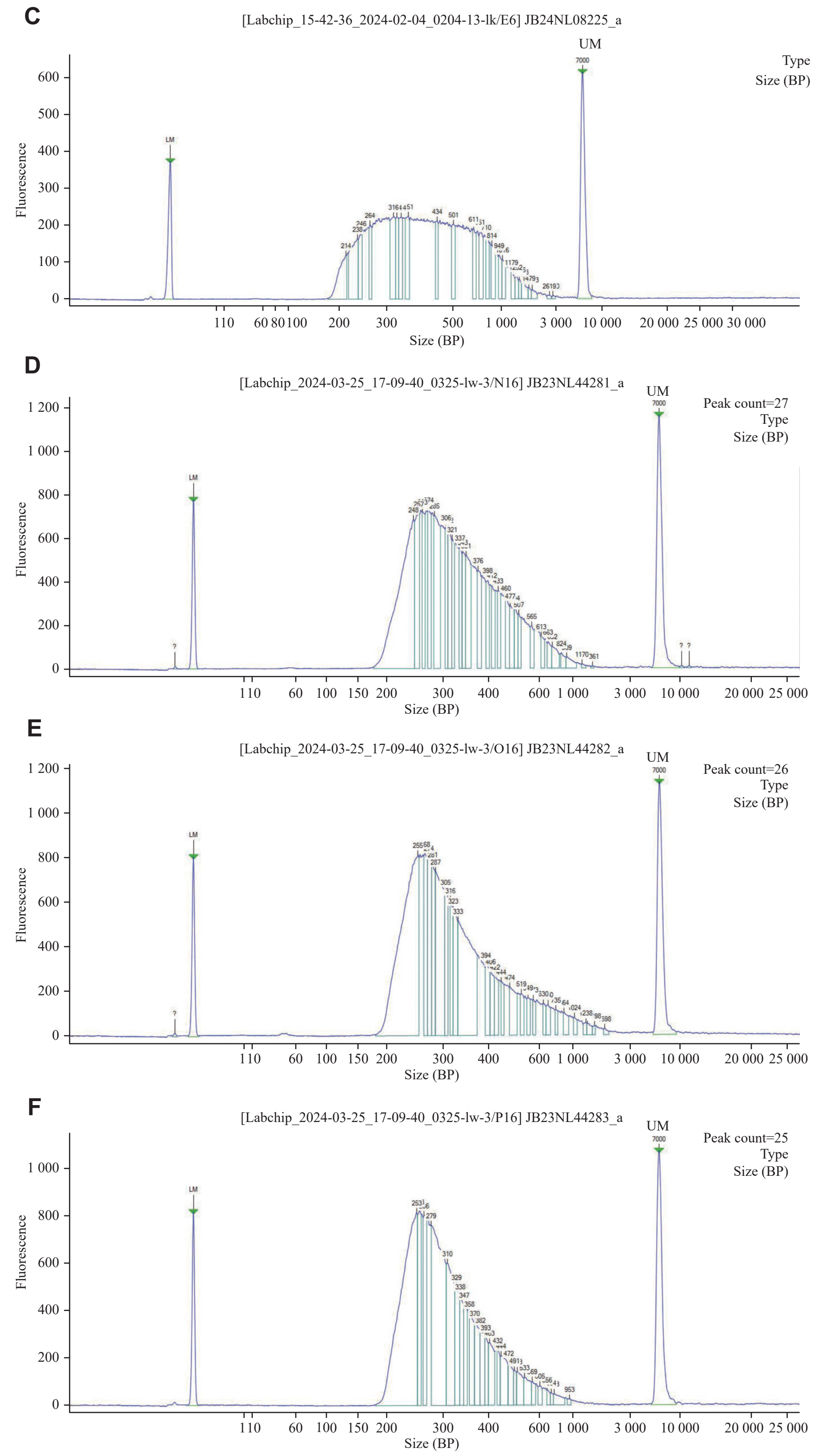


***Supplementary Fig. 1*** **The construction of circular RNA libraries using the SHERRY method.** Testicular (A–C) and seminal plasma (D–F) circular RNA sequencing quality control peak.

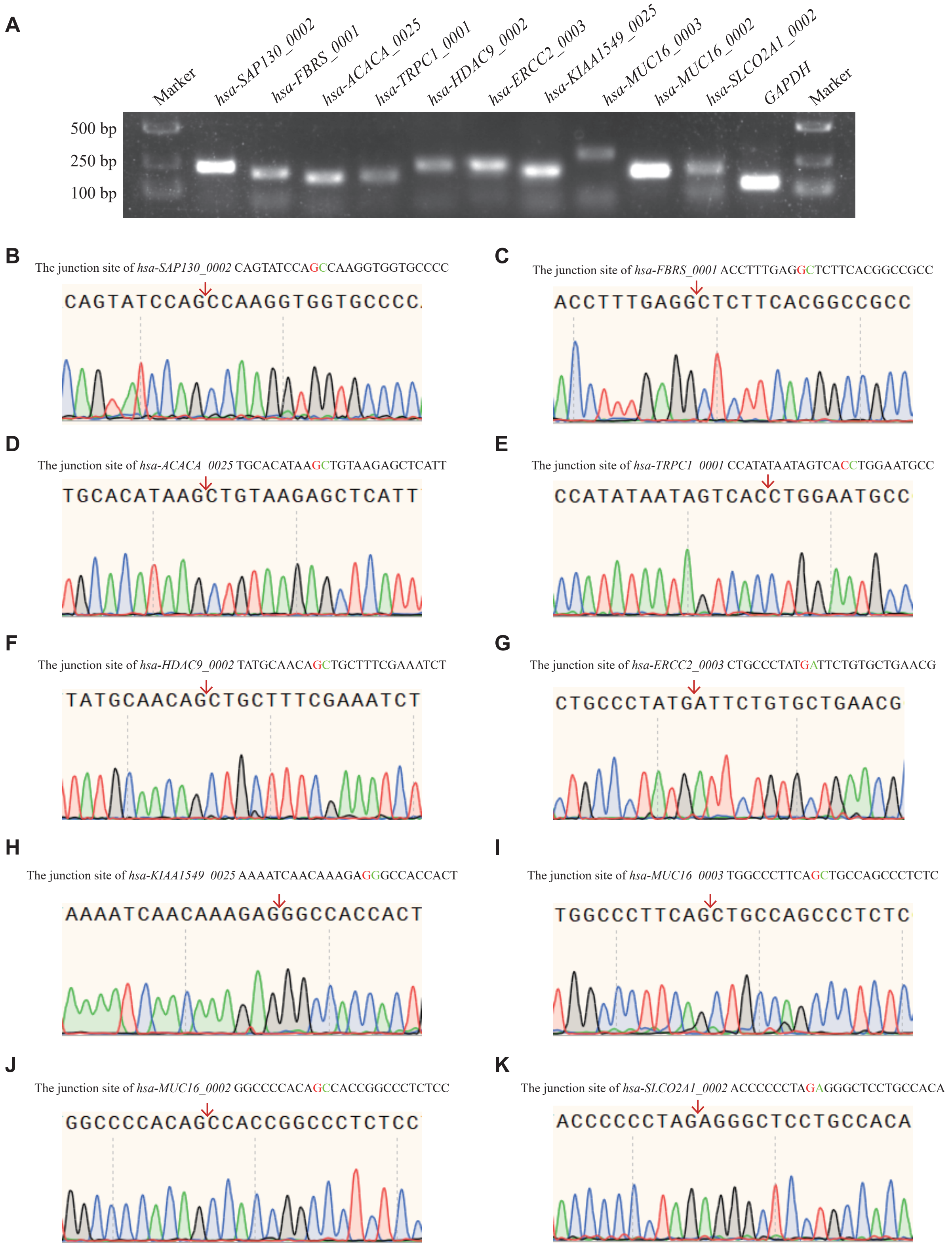


***Supplementary Fig. 2*** **Validation of 10 circRNAs.** A: Agarose gel electrophoresis image of circRNA PCR products. B–K: Sanger sequencing results of PCR products of *hsa-SAP130_0002*, *hsa-FBRS_0001*, *hsa-ACACA_0025*, *hsa-TRPC1_0001*, *hsa-HDAC9_0002*, *hsa-ERCC2_0003*, *hsa-KIAA1549_0025*, *hsa-MUC16_0003*, *hsa-MUC16_0002*, *hsa-SLCO2A1_0002*, and *GAPDH*.

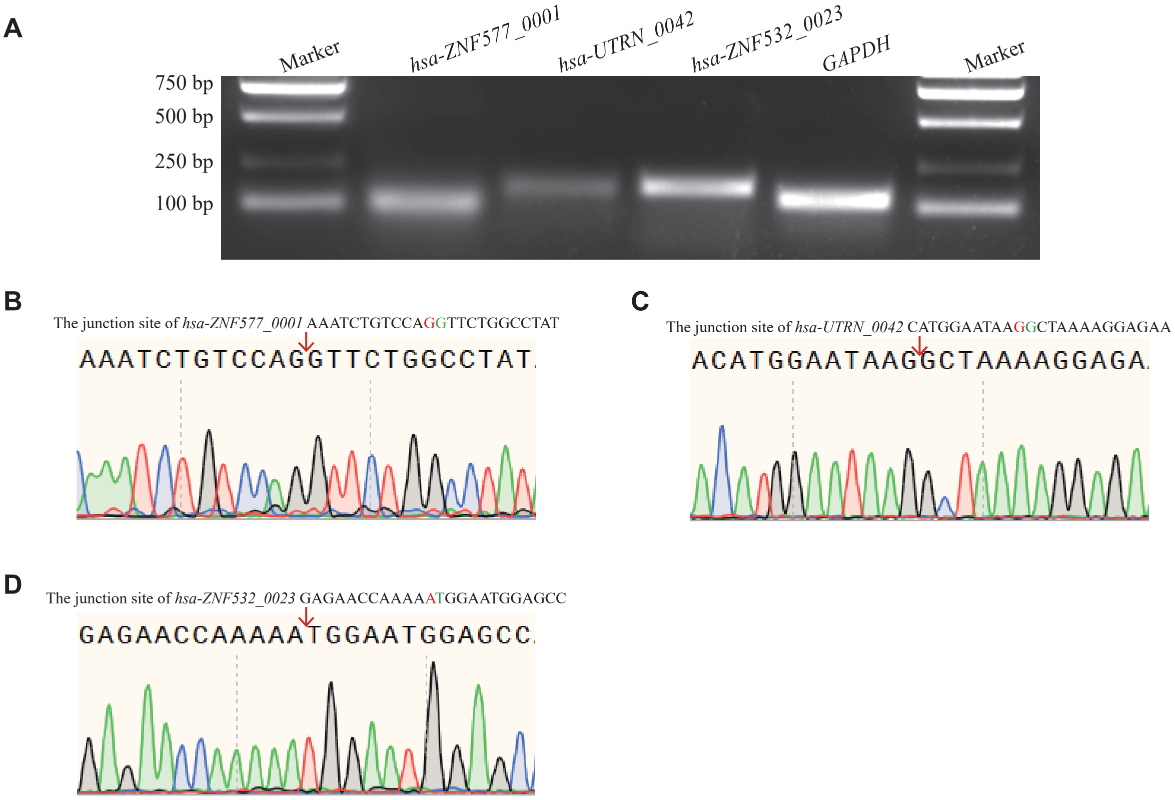


***Supplementary Fig. 3*** **Validation of three circRNAs.** A: Agarose gel electrophoresis image of circRNA PCR products. B–D: Sanger sequencing results of PCR products of *hsa-ZNF577_0001*, *hsa-UTRN_0042*, *hsa-ZNF532_0023*, and *GAPDH*.

***Supplementary Table 1*** **circRNA primer sequence**

| circRNAs | Position | Primer (5′-3′) | PCR product size (bp) |
|---|---|---|---|
| *hsa-SAP130_0002* | chr2:127993187:127996491:− | Forward: ATCCGACAGTATCCAGCCAA<br>Reverse: CAGTGGGTATGCAGAAGGGG | 222 |
| *hsa-FBRS_0001* | chr16:30664215:30666541:+ | Forward: CGGCCACCTTTGAGGCTCTT<br>Reverse: GGGAAAGCTGTGGGTGGACAA | 184 |
| *hsa-ACACA_0025* | chr17:37330173:37330425:− | Forward: GGCCTTGCACATAAGCTGT<br>Reverse: GTCCAACTTCACCAGGTTGCT | 161 |
| *hsa-TRPC1_0001* | chr3:142736379:142748460:+ | Forward: ACAACCGTAGTCCAAAAGAAGC<br>Reverse: TCCGGCATTCCAGGTGACTA | 165 |
| *hsa-HDAC9_0002* | chr7:18666213:18666476:+ | Forward: CGATGCAGGAAGACAGAGCG<br>Reverse: TGTTCAATAGATTTCGAAAGCAGC | 224 |
| *hsa-ERCC2_0003* | chr19:45357270:45361642:− | Forward: CGCTGGACATCTACCCCAAG<br>Reverse: ATGGTGAAGCCTTTGGCGTA | 221 |
| *hsa-KIAA1549_0005* | chr7:138871157:138871362:− | Forward: ATCTTCGAGCACGTGGACAG<br>Reverse: CTCATTTCCCGAACTGGGCA | 188 |
| *hsa-MUC16_0003* | chr19:8884959:8886860:− | Forward: CCTGGACAACGACAGCCTCT<br>Reverse: CCAACACTGGTGCTCTTGAACA | 288 |
| *hsa-MUC16_0002* | chr19:8894193:8896090:− | Forward: GACTTTCACAGTACAGCCGGA<br>Reverse: GAGCAGACCCTGCAGAACAC | 187 |
| *hsa-SLCO2A1_0002* | chr3:133947256:133948971:− | Forward: CCAACTGTGGCCGAAGTCTACC<br>Reverse: GCCAGCAATGACGGAGGAGAAG | 200 |
| *hsa-ZNF577_0001* | chr19:51877282:51880877:− | Forward: ACCAAGCCAGATTCGCTCTT<br>Reverse: TCCTATAGGCCAGAACCTGGA | 102 |
| *hsa-UTRN_0042* | chr6:144537582:144539443:+ | Forward: TGTAAATATGACATGGAATAAGGCT<br>Reverse: TCTTAATTCTTGCAGGTTTTCTCC | 158 |
| *hsa-ZNF532_0023* | chr18:58931646:58939621:+ | Forward: ATGTGGCTGCTCTGAAGTCTC<br>Reverse: TGCTGGCTCCATTCCATTTTTG | 163 |
| *GAPDH* | | Forward: GGCCTCCAAGGAGTAAGACC<br>Reverse: AGGGGAGATTCAGTGTGGTG | 122 |

**Supplementary Table 2** ceRNA network interaction

| Node1 | Node2 | Node1 | Node2 | Node1 | Node2 | Node1 | Node2 |
|---|---|---|---|---|---|---|---|
| hsa-TRPC1_0001 | hsa-miR-150-5p | hsa-miR-7848-3p | SPRY4 | hsa-miR-4776-3p | TRPS1 | hsa-miR-4640-3p | FOXK1 |
| hsa-SAP130_0002 | hsa-miR-7848-3p | hsa-miR-7848-3p | RORA | hsa-miR-4776-3p | TRIM14 | hsa-miR-4640-3p | EID1 |
| hsa-SAP130_0002 | hsa-miR-6798-3p | hsa-miR-7848-3p | MIER3 | hsa-miR-4776-3p | TCN2 | hsa-miR-4640-3p | EDEM3 |
| hsa-SAP130_0002 | hsa-miR-584-3p | hsa-miR-7848-3p | BCL2L11 | hsa-miR-4776-3p | TBCEL | hsa-miR-4640-3p | E2F7 |
| hsa-SAP130_0002 | hsa-miR-4776-3p | hsa-miR-6798-3p | ZSCAN16 | hsa-miR-4776-3p | SPRY4 | hsa-miR-4640-3p | DNAAF2 |
| hsa-SAP130_0002 | hsa-miR-4758-3p | hsa-miR-6798-3p | ZNF449 | hsa-miR-4776-3p | SEC14L4 | hsa-miR-4640-3p | CHST15 |
| hsa-SAP130_0002 | hsa-miR-4640-3p | hsa-miR-6798-3p | TMEM245 | hsa-miR-4776-3p | PAK5 | hsa-miR-4640-3p | CD226 |
| hsa-SAP130_0002 | hsa-miR-4436b-5p | hsa-miR-6798-3p | TM4SF1 | hsa-miR-4776-3p | MTMR9 | hsa-miR-4640-3p | BBC3 |
| hsa-SAP130_0002 | hsa-miR-323a-5p | hsa-miR-6798-3p | SMIM12 | hsa-miR-4776-3p | LRRC58 | hsa-miR-4640-3p | ARL9 |
| hsa-SAP130_0002 | hsa-miR-150-5p | hsa-miR-6798-3p | PNPLA6 | hsa-miR-4776-3p | LRAT | hsa-miR-4640-3p | ARL5C |
| hsa-SAP130_0002 | hsa-miR-127-3p | hsa-miR-6798-3p | MAF | hsa-miR-4776-3p | HYPK | hsa-miR-4436b-5p | TMED4 |
| hsa-miR-92a-2-5p | ZNF385A | hsa-miR-6798-3p | ISLR2 | hsa-miR-4776-3p | GTF2A1 | hsa-miR-4436b-5p | TBCEL |
| hsa-miR-92a-2-5p | UBE2V1 | hsa-miR-6798-3p | GOLGA7B | hsa-miR-4776-3p | ETS1 | hsa-miR-4436b-5p | SYNJ2BP |
| hsa-miR-92a-2-5p | TFAP2B | hsa-miR-6798-3p | GOLGA1 | hsa-miR-4776-3p | EBF1 | hsa-miR-4436b-5p | SMCR8 |
| hsa-miR-92a-2-5p | SLIT1 | hsa-miR-6798-3p | GCDH | hsa-miR-4776-3p | DPY19L1 | hsa-miR-4436b-5p | SLC1A5 |
| hsa-miR-92a-2-5p | SKI | hsa-miR-6798-3p | EID1 | hsa-miR-4776-3p | BICC1 | hsa-miR-4436b-5p | SETD5 |
| hsa-miR-92a-2-5p | RNF40 | hsa-miR-6798-3p | EDEM3 | hsa-miR-4776-3p | AGO3 | hsa-miR-4436b-5p | PPM1A |
| hsa-miR-92a-2-5p | RHOA | hsa-miR-6798-3p | E2F7 | hsa-miR-4776-3p | ABCG8 | hsa-miR-4436b-5p | PDCD4 |
| hsa-miR-92a-2-5p | PPP1R9B | hsa-miR-6798-3p | DNAAF2 | hsa-miR-4776-3p | ZFP91 | hsa-miR-4436b-5p | MPPE1 |
| hsa-miR-92a-2-5p | POLR2E | hsa-miR-6798-3p | CHST15 | hsa-miR-4758-3p | ZFP91 | hsa-miR-4436b-5p | MIER3 |
| hsa-miR-92a-2-5p | PLEKHM1 | hsa-miR-6798-3p | CD226 | hsa-miR-4758-3p | TNFAIP8L1 | hsa-miR-4436b-5p | LRAT |
| hsa-miR-92a-2-5p | NKX2-2 | hsa-miR-6798-3p | ARL9 | hsa-miR-4758-3p | SLC35F6 | hsa-miR-4436b-5p | LIPG |
| hsa-miR-92a-2-5p | NFIX | hsa-miR-6798-3p | ARL5C | hsa-miR-4758-3p | MKNK2 | hsa-miR-4436b-5p | LAIR1 |
| hsa-miR-92a-2-5p | NFIC | hsa-miR-584-3p | ZNF582 | hsa-miR-4758-3p | KMT2D | hsa-miR-4436b-5p | GJD3 |
| hsa-miR-92a-2-5p | NACC1 | hsa-miR-584-3p | ZNF460 | hsa-miR-4758-3p | GTF2A1 | hsa-miR-4436b-5p | DMRT2 |
| hsa-miR-92a-2-5p | MTA1 | hsa-miR-584-3p | SMCR8 | hsa-miR-4758-3p | FADS1 | hsa-miR-4436b-5p | COX19 |
| hsa-miR-92a-2-5p | MEX3A | hsa-miR-584-3p | SKI | hsa-miR-4758-3p | DPY19L1 | hsa-miR-4436b-5p | BASP1 |
| hsa-miR-92a-2-5p | MAP2K3 | hsa-miR-584-3p | SETD5 | hsa-miR-4758-3p | CCND1 | hsa-miR-323a-5p | ULK2 |
| hsa-miR-92a-2-5p | GTPBP1 | hsa-miR-584-3p | RANBP1 | hsa-miR-4758-3p | ANKRD65 | hsa-miR-323a-5p | TTLL12 |
| hsa-miR-92a-2-5p | GIPC1 | hsa-miR-584-3p | PURB | hsa-miR-4640-3p | ZSCAN16 | hsa-miR-323a-5p | TNIP3 |
| hsa-miR-92a-2-5p | FOSL1 | hsa-miR-584-3p | POTEM | hsa-miR-4640-3p | ZNF449 | hsa-miR-323a-5p | TMED4 |
| hsa-miR-92a-2-5p | FIBCD1 | hsa-miR-584-3p | POTEG | hsa-miR-4640-3p | TRIM14 | hsa-miR-323a-5p | SYNPO2L |
| hsa-miR-92a-2-5p | FGF19 | hsa-miR-584-3p | MKNK2 | hsa-miR-4640-3p | TMEM245 | hsa-miR-323a-5p | RPL14 |
| hsa-miR-92a-2-5p | DOT1L | hsa-miR-584-3p | HNRNPU | hsa-miR-4640-3p | TM4SF1 | hsa-miR-323a-5p | POTEM |
| hsa-miR-92a-2-5p | CELSR2 | hsa-miR-584-3p | GTF2A1 | hsa-miR-4640-3p | SMIM12 | hsa-miR-323a-5p | POTEG |
| hsa-miR-92a-2-5p | CELF5 | hsa-miR-584-3p | ETS1 | hsa-miR-4640-3p | SLC7A11 | hsa-miR-323a-5p | KPNA6 |
| hsa-miR-92a-2-5p | CBX6 | hsa-miR-584-3p | CRAMP1 | hsa-miR-4640-3p | SCN2B | hsa-miR-323a-5p | ENTPD4 |
| hsa-miR-92a-2-5p | CAMK2A | hsa-miR-584-3p | BCL2L11 | hsa-miR-4640-3p | PNPLA6 | hsa-miR-323a-5p | DMRT2 |
| hsa-miR-92a-2-5p | BCL7A | hsa-miR-4776-3p | ZNF99 | hsa-miR-4640-3p | MAF | hsa-miR-323a-5p | CRAMP1 |
| hsa-miR-92a-2-5p | BAZ1B | hsa-miR-4776-3p | ZNF786 | hsa-miR-4640-3p | ISLR2 | hsa-miR-323a-5p | CCND1 |
| hsa-miR-92a-2-5p | AR | hsa-miR-4776-3p | ZNF708 | hsa-miR-4640-3p | GOLGA7B | hsa-miR-323a-5p | CAPZA2 |
| hsa-miR-92a-2-5p | APH1A | hsa-miR-4776-3p | ZNF208 | hsa-miR-4640-3p | GOLGA1 | hsa-miR-323a-5p | BBC3 |
| hsa-miR-7848-3p | TGFBR3 | hsa-miR-4776-3p | ZNF138 | hsa-miR-4640-3p | GCDH | hsa-miR-323a-5p | ATP1B3 |

***Supplementary Table 2*** **ceRNA network interaction** (continued)

| Node1 | Node2 | Node1 | Node2 | Node1 | Node2 | Node1 | Node2 |
|---|---|---|---|---|---|---|---|
| hsa-miR-323a-5p | ANKFY1 | hsa-miR-150-5p | COX19 | hsa-miR-128-1-5p | NKX2-2 | hsa-miR-8052 | PRDM6 |
| hsa-miR-323a-5p | AMD1 | hsa-miR-150-5p | CBL | hsa-miR-128-1-5p | NFIX | hsa-miR-8052 | GAB2 |
| hsa-miR-323a-5p | ABHD18 | hsa-miR-150-5p | CAPZA2 | hsa-miR-128-1-5p | MAP2K3 | hsa-miR-8052 | SYNPO2L |
| hsa-miR-150-5p | ZNF786 | hsa-miR-150-5p | BASP1 | hsa-miR-128-1-5p | GIPC1 | hsa-miR-8052 | SULT1C2 |
| hsa-miR-150-5p | ZNF708 | hsa-miR-150-5p | ATP1B3 | hsa-miR-128-1-5p | DOT1L | hsa-miR-4446-3p | DR1 |
| hsa-miR-150-5p | ZNF582 | hsa-miR-150-5p | ANKRD65 | hsa-miR-128-1-5p | CBX6 | hsa-miR-4446-3p | CBX7 |
| hsa-miR-150-5p | ZNF460 | hsa-miR-150-5p | ANKFY1 | hsa-miR-127-3p | SKI | hsa-miR-4446-3p | MBNL2 |
| hsa-miR-150-5p | ZFP91 | hsa-miR-150-5p | AMD1 | hsa-FBRS_0001 | hsa-miR-92a-2-5p | hsa-miR-4446-3p | PHF24 |
| hsa-miR-150-5p | VPS53 | hsa-miR-150-5p | AGO3 | hsa-FBRS_0001 | hsa-miR-138-5p | hsa-miR-4446-3p | CDK18 |
| hsa-miR-150-5p | ULK2 | hsa-miR-150-5p | ADIPOR2 | hsa-FBRS_0001 | hsa-miR-128-1-5p | hsa-miR-4446-3p | BRCA1 |
| hsa-miR-150-5p | TTLL12 | hsa-miR-150-5p | ABHD18 | hsa-UTRN_0042 | hsa-miR-577 | hsa-miR-4446-3p | C1orf162 |
| hsa-miR-150-5p | TRPS1 | hsa-miR-150-5p | ABCG8 | hsa-UTRN_0042 | hsa-miR-216a-5p | hsa-miR-6878-5p | VGLL3 |
| hsa-miR-150-5p | TNIP3 | hsa-miR-138-5p | ZNF385A | hsa-UTRN_0042 | hsa-miR-1183 | hsa-miR-6878-5p | CELF3 |
| hsa-miR-150-5p | TNFAIP8L1 | hsa-miR-138-5p | ZEB2 | hsa-UTRN_0042 | hsa-miR-3169 | hsa-miR-6878-5p | CNOT4 |
| hsa-miR-150-5p | TCN2 | hsa-miR-138-5p | VIM | hsa-UTRN_0042 | hsa-miR-8052 | hsa-miR-6878-5p | MTCH2 |
| hsa-miR-150-5p | SYNPO2L | hsa-miR-138-5p | UBE2V1 | hsa-UTRN_0042 | hsa-miR-4446-3p | hsa-miR-6788-5p | AKAP6 |
| hsa-miR-150-5p | SYNJ2BP | hsa-miR-138-5p | RMND5A | hsa-UTRN_0042 | hsa-miR-6878-5p | hsa-miR-6788-5p | MARCKSL1 |
| hsa-miR-150-5p | SP1 | hsa-miR-138-5p | RELN | hsa-UTRN_0042 | hsa-miR-6788-5p | hsa-miR-6788-5p | MDGA2 |
| hsa-miR-150-5p | SLC7A11 | hsa-miR-138-5p | RARA | hsa-UTRN_0042 | hsa-miR-3173-3p | hsa-miR-6788-5p | RASSF8 |
| hsa-miR-150-5p | SLC35F6 | hsa-miR-138-5p | POLR2E | hsa-UTRN_0042 | hsa-miR-6884-5p | hsa-miR-6788-5p | KRT222 |
| hsa-miR-150-5p | SLC1A5 | hsa-miR-138-5p | PLEKHM1 | hsa-miR-577 | GOLPH3 | hsa-miR-6788-5p | PELI1 |
| hsa-miR-150-5p | SEC14L4 | hsa-miR-138-5p | NFIX | hsa-miR-577 | DAZ1 | hsa-miR-3173-3p | PLEKHS1 |
| hsa-miR-150-5p | SCN2B | hsa-miR-138-5p | NACC1 | hsa-miR-577 | DAZ4 | hsa-miR-3173-3p | OXSR1 |
| hsa-miR-150-5p | RPL14 | hsa-miR-138-5p | MEX3A | hsa-miR-577 | NEK1 | hsa-miR-3173-3p | UBE2Q1 |
| hsa-miR-150-5p | PURB | hsa-miR-138-5p | GTPBP1 | hsa-miR-577 | DAZ3 | hsa-miR-3173-3p | ARID1A |
| hsa-miR-150-5p | PRKCA | hsa-miR-138-5p | FOXC1 | hsa-miR-577 | INO80D | hsa-miR-3173-3p | PPP6C |
| hsa-miR-150-5p | PPM1A | hsa-miR-138-5p | FOSL1 | hsa-miR-216a-5p | DMXL2 | hsa-miR-6884-5p | KCNB1 |
| hsa-miR-150-5p | PDCD4 | hsa-miR-138-5p | FGF19 | hsa-miR-216a-5p | TMEM161B | hsa-miR-6884-5p | PLXNA4 |
| hsa-miR-150-5p | MYB | hsa-miR-138-5p | FERMT2 | hsa-miR-216a-5p | BOD1L1 | hsa-miR-6884-5p | GPN3 |
| hsa-miR-150-5p | MTMR9 | hsa-miR-138-5p | EID1 | hsa-miR-216a-5p | HOOK1 | hsa-miR-6884-5p | PHTF2 |
| hsa-miR-150-5p | MPPE1 | hsa-miR-138-5p | DNAJB6 | hsa-miR-216a-5p | KLF9 | hsa-ACACA_0025 | hsa-miR-212-5p |
| hsa-miR-150-5p | LRRC58 | hsa-miR-138-5p | DEK | hsa-miR-1183 | XRN2 | hsa-miR-212-5p | RNF185 |
| hsa-miR-150-5p | LIPG | hsa-miR-138-5p | CELF5 | hsa-miR-1183 | IKZF2 | hsa-miR-212-5p | SSRP1 |
| hsa-miR-150-5p | LAIR1 | hsa-miR-138-5p | CCND3 | hsa-miR-1183 | RFX7 | hsa-miR-212-5p | NPEPPS |
| hsa-miR-150-5p | KPNA6 | hsa-miR-138-5p | CASTOR2 | hsa-miR-1183 | RASSF8 | hsa-miR-212-5p | CDK2 |
| hsa-miR-150-5p | HYPK | hsa-miR-138-5p | BAZ1B | hsa-miR-1183 | KLF3 | hsa-miR-212-5p | GNB1L |
| hsa-miR-150-5p | HILPDA | hsa-miR-138-5p | ARHGAP42 | hsa-miR-1183 | PTPRD | hsa-miR-212-5p | IL1F10 |
| hsa-miR-150-5p | GJD3 | hsa-miR-138-5p | AGO1 | hsa-miR-1183 | PPP2CA | hsa-miR-212-5p | SLC47A1 |
| hsa-miR-150-5p | FOXK1 | hsa-miR-128-1-5p | TFAP2B | hsa-miR-1183 | ERRFI1 | hsa-miR-212-5p | C1D |
| hsa-miR-150-5p | FADS1 | hsa-miR-128-1-5p | SKI | hsa-miR-3169 | SLC22A1 | hsa-miR-212-5p | NFIX |
| hsa-miR-150-5p | EPHB2 | hsa-miR-128-1-5p | RNF40 | hsa-miR-3169 | SPG11 | hsa-miR-212-5p | RASL10B |
| hsa-miR-150-5p | ENTPD4 | hsa-miR-128-1-5p | RHOA | hsa-miR-3169 | ELOVL6 | | |
